# Modeling Alcohol Dehydrogenase Catalysis in Deep Eutectic Solvent/Water Mixtures

**DOI:** 10.1002/cbic.201900624

**Published:** 2019-12-13

**Authors:** Lei Huang, Jan Philipp Bittner, Pablo Domínguez de María, Sven Jakobtorweihen, Selin Kara

**Affiliations:** ^1^ Department of Engineering Biocatalysis and Bioprocessing Group Aarhus University Gustav Wieds Vej 10 8000 Aarhus Denmark; ^2^ Institute of Thermal Separation Processes Hamburg University of Technology Eißendorfer Strasse 38 21073 Hamburg Germany; ^3^ Sustainable Momentum S.L. Av. Ansite 3, 4–6 35011 Las Palmas de Gran Canaria Canary Islands Spain

**Keywords:** alcohol dehydrogenase, deep eutectic solvents, molecular dynamics, solvent effects, thermodynamics

## Abstract

The use of oxidoreductases (EC1) in non‐conventional reaction media has been increasingly explored. In particular, deep eutectic solvents (DESs) have emerged as a novel class of solvents. Herein, an in‐depth study of bioreduction with an alcohol dehydrogenase (ADH) in the DES glyceline is presented. The activity and stability of ADH in mixtures of glyceline/water with varying water contents were measured. Furthermore, the thermodynamic water activity and viscosity of mixtures of glyceline/water have been determined. For a better understanding of the observations, molecular dynamics simulations were performed to quantify the molecular flexibility, hydration layer, and intraprotein hydrogen bonds of ADH. The behavior of the enzyme in DESs follows the classic dependence of water activity (*a*_W_) in non‐conventional media. At low *a*_W_ values (<0.2), ADH does not show any activity; at higher *a*_W_ values, the activity was still lower than that in pure water due to the high viscosities of the DES. These findings could be further explained by increased enzyme flexibility with increasing water content.

## Graphical abstract

**Enzyme solvation**: The use of deep eutectic solvents (DESs) is examined experimentally and computationally for the alcohol dehydrogenase catalyzed reaction. The outcomes of molecular dynamics simulations complement experimental observations on catalytic performance of the enzyme for a greater understanding of influencing factors.

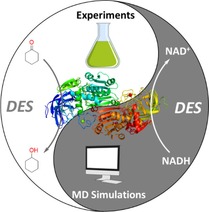

## Introduction

Oxidoreductases (EC1) are the most commonly applied biocatalysts, following hydrolases (EC3), for the synthesis of active pharmaceutical intermediates (APIs) and fine chemicals on both academic and industrial scales.^1^ The use of nonaqueous media for oxidoreductases has been of high interest over the past decades, providing alternative solutions for achieving high volumetric productivities and product titers, as well as for overcoming water‐related limitations.^2^ The nonaqueous media that have attracted great attention for redox biocatalysis can be classified into three main categories: neat substrate systems, organic solvents, and deep eutectic solvents (DESs).2a More classical neat substrate and organic solvent systems are both widely applied for EC3, but DESs have emerged as a new type of solvent in (redox) biocatalysis, owing to their tunable properties, nontoxicity, biodegradability, and lower cost.^3^

**Figure 1:**
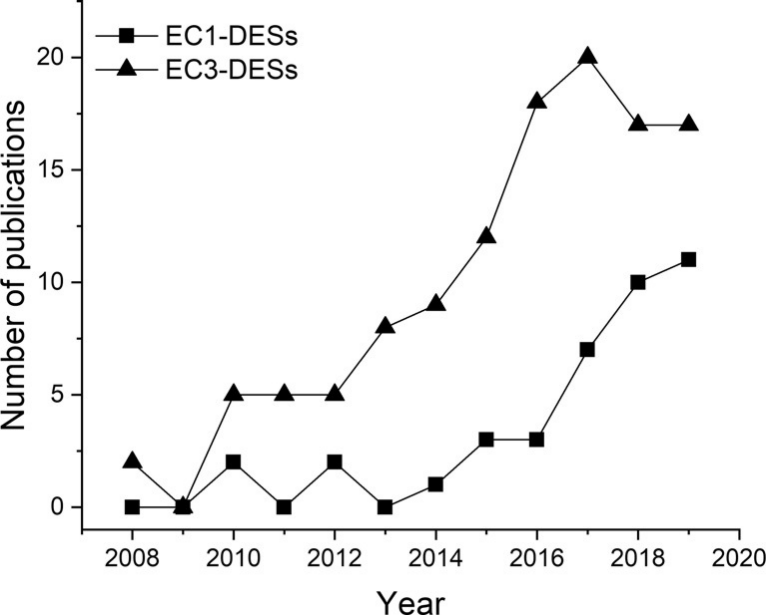
Number of publications on DESs in the biocatalysis field of EC1 (oxidoreductases) and EC3 (hydrolases) in the last decade (source: Web of Science, up to August 1, 2019).

A DES is typically formed by the combination of a hydrogen‐bond acceptor (HBA)—in most cases, a quaternary ammonium salt, such as choline chloride (ChCl)—and a hydrogen‐bond donor (HBD), such as polyols, carboxylic acids, and amines, at a certein molar ratio (e.g., HBA/HBD of 1:2).^4^ The hydrogen‐bond association between the HBA and HBD disrupts the crystalline structures of individual components, forming liquids at room temperature, which permits DESs to be used as liquid reaction media for biocatalysis.^5^ The use of DESs as media for biocatalysis has been widely explored in the field of hydrolases (EC3), particularly lipase‐catalyzed esterification, transesterification, aminolysis, and epoxidation reactions.^6^ Herein, the application of EC1 in DESs is also gaining importance,^7^ as observed in publications related to EC1‐DESs and EC3‐DESs reported during the past decade (Figure 1). Given the high potential of DESs, other enzyme classes, for example, lyases (EC4), have been recently documented as well.^8^

To date, in the field of redox biocatalysis, DESs have been explored by using whole cells7e, 9 and isolated ketoreductases (KREDs)^10^ and alcohol dehydrogenases (ADHs),^11^ as well as in bio‐oxidations mediated by ADHs,^12^ heme‐dependent enzymes,7b, 13 laccases,^14^ or catalases.7f, 15 Due to the good biocompatibility of DESs and their promotion of increased cell permeability, redox reactions catalyzed by whole cells show higher conversion and stability.7e, 9f, 12, 16 Moreover, DESs display beneficial effects, or even stereoinversion, on the enantioselectivity of bioreduction catalyzed by baker's yeast cells.9a, 9e Some isolated enzymes (mainly ADHs) also show good performance and the ability to be combined with metal catalysts in DESs.^10^, ^11^, ^17^ However, a rational evaluation of the effect of DESs, as non‐conventional media, on the behavior of oxidoreductases is still missing. To shed some light on these aspects, herein, we evaluate the empirical performance of pure ADHs in mixtures of DES–water, and compare it with theoretical calculations and classical parameters, such as water activity.

Molecular dynamics (MD) simulations can provide great opportunities for a deeper understanding of the interactions between enzymes, solvents, and water. In recent years, MD simulations have been used to create a detailed picture of solvent–protein interactions, as well as to explain experimentally observed behavior, mainly of lipases (EC3).^18^ Initial investigations focused mainly on enzyme‐bound water because the hydration layer was essential for the catalytic activity.^19^ Thus, by analyzing water clusters on the surface of *Candida antarctica* lipase B (CALB), the preferential binding sites of water were found to be independent of the organic solvent, although the hydration level was largely influenced by the solvent and its capacity to withdraw water molecules.18b, 18c Moreover, no evidence of a complete water layer around the enzymes was found and some parts of the protein were in direct contact with the organic solvent, even at large water contents.18b The thermodynamic activity of the solvent molecules in the bulk phase are essential factors for enzymatic catalysis,18d, 18f since the bulk‐phase water activity (*a*_W_), for example, defines the hydration layer of CALB.18d Apart from hydration of the enzyme, its conformational flexibility is a crucial factor for catalytic activity. For instance, in a study of CALB in low‐water organic media, Trodler and Pleiss related the octanol–water partition coefficients (log *P*) of five organic solvents to the averaged *B* factors of CALB, whereby a lower log *P* was associated with increased enzyme flexibility.18a

Regarding the novel solvent category of DESs, to the best of our knowledge, to date, only a study by Monhemi et al. reported the interactions of DES with an enzyme by using MD simulations,18e describing stabilization of CALB in the DES comprised of ChCl and urea, although urea usually causes unfolding of proteins.6a Through MD simulations, it was found that hydrogen bonds between urea molecules and choline and chloride ions resulted in urea with a low diffusion coefficient that could not reach the enzyme domains.18e

To achieve a deeper understanding the effects of DESs on oxidoreductases, which ultimately may expand the applicability of DESs, herein we assess the behavior of an EC1 enzyme in a chosen DES system with the support of MD simulations. Thus, we selected one of the earliest and best characterized oxidoreductases, ADH isolated from horse liver (HLADH; EC 1.1.1.1),^20^ the EE‐isozyme of which has been widely studied for its application in nonaqueous media.^21^ A eutectic mixture of ChCl and glycerol (Gly) (1:2 mol mol^−1^), which is also known as glyceline, was selected as a prototypical DES for ADH‐catalyzed reactions. Glyceline is one of the most successfully applied DESs for biocatalysis.6b, 8a, 9c, 10, 12a, 17 MD simulations focus on the quantitative analysis of protein flexibility and hydration level, whereby the results from enzymatic activity and stability are correlated and explained.

## Results and Discussion

**Scheme 1:**
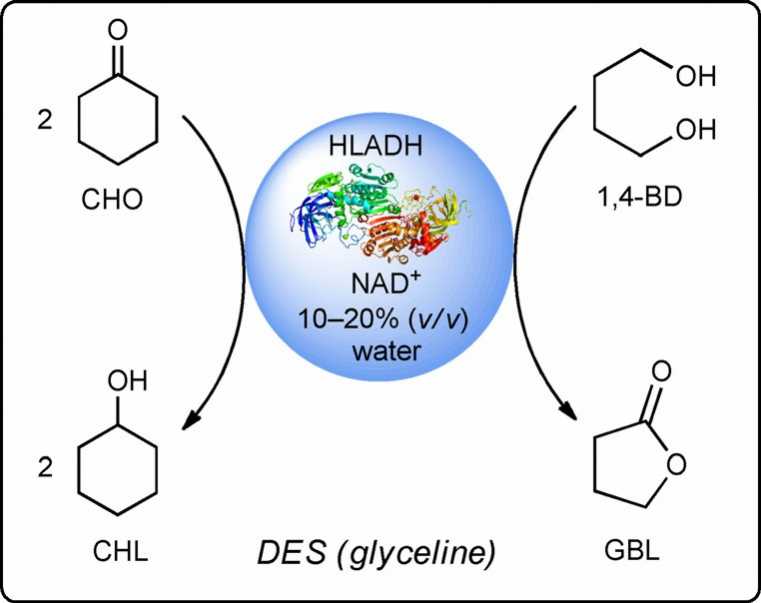
Reduction of CHO to CHL, as catalyzed by HLADH, promoted by 1,4‐BD in mixtures of glyceline/water as reaction media. NAD: nicotinamide adenine dinucleotide.

As model reaction, we selected the HLADH‐catalyzed bioreduction of cyclohexanone (CHO) to cyclohexanol (CHL) coupled with butane‐1,4‐diol (1,4‐BD), which was a “smart cosubstrate” for cofactor regeneration (Scheme 1). The formation of thermodynamically stable and kinetically inert coproduct, γ‐butyrolactone, can make the regeneration reaction irreversible, thus simplifying the theoretical analysis of the enzymatic performance.^22^

As stated above, water is essential for the catalytic activity of enzymes. Although enzymes can be successfully applied in nonaqueous systems—in the absence of bulk water—an enzyme‐bound essential layer of water is needed to maintain sufficient conformational flexibility for catalysis.^23^ The exact amount of water molecules needed for an enzyme to be catalytically active is highly enzyme specific. An indirect quantification of the amount of bound water is possible because it is influenced by the thermodynamic water activity (*a*_w_) of the medium and has been used as the most suitable parameter to measure and control the amount of water in organic media.18d, 24 Different enzymes have different water requirements for optimal activity, and hence, the *a*_w_ values have to be adjusted to meet the requirements of the enzyme.

**Figure 2:**
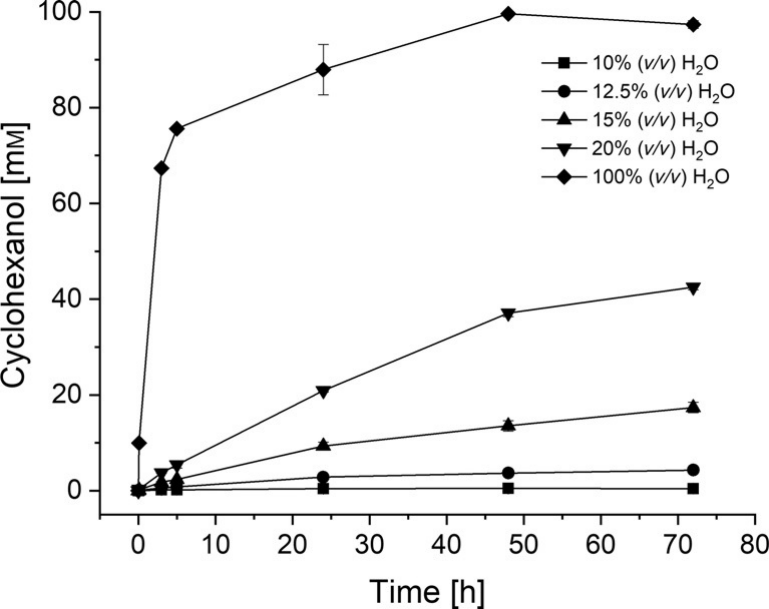
HLADH‐catalyzed reduction of CHO in various mixtures of glyceline/H_2_O for 72 h. Reaction conditions: 100 mm CHO, 50 mm 1,4‐BD, 1 mm NAD^+^, 1 mg mL^−1^ purified HLADH in glyceline/water at 25 °C and 1200 rpm. Buffer (50 mm Tris**⋅**HCl, pH 7.5) was added to incubate the enzyme with NAD^+^. The data points are connected by a solid line to guide the eye. Results are from duplicate experiments with a maximum standard deviation of 5 %.

On that basis, the model reaction was assessed in the mixtures of glyceline/water with various water contents, ranging from 0 to 20 % (*v*/*v*; Figure 2). A reaction was performed in water (100 % *v*/*v*, 50 mm Tris**⋅**HCl, pH 7.5 buffer) for comparison.

The purified HLADH in the presence of various mixtures of glyceline/water displayed much lower product yields at 72 h (ranging from 0.5 to 43 %) compared with the yield (97 %) achieved in the pure buffer system. There was no product detected in the systems with less than 10 % H_2_O (*v*/*v*; i.e., 0, 1.36, and 5 %), presumably due to enzyme deactivation caused by the deficiency of water to maintain the conformational flexibility of HLADH for catalysis and/or potential enzyme destabilization under low‐water conditions. In a previous study, whole cells of *Escherichia coli* containing overexpressed HLADH could achieve approximate 80 % product yield in glyceline with 20 % H_2_O (*v*/*v*), which was two times higher than that in the case of free enzyme.9c This is consistent with other reported whole‐cell biocatalysis in mixtures of DES/water, since the integrity of the cells secures the stability of enzymes, relative to a totally pure free enzyme.9a, 9b, 9e

As mentioned before, the enzyme activity mainly depends on enzyme‐bound water, which is ultimately influenced by the thermodynamic *a*_w_ value.^25^ Therefore, the *a*_w_ values of these mixtures of glyceline/water were determined (Figure S3 in the Supporting Information). Overall, the *a*_W_ value of glyceline increased gradually with increasing water content, up to 20 % (*v*/*v*), which was in agreement with a previous report by Wu et al.^26^ This implies that the *a*_w_ of glyceline can be tailored in a controlled way by the addition of water. Product formation could only be detected in mixtures of glyceline/water with ≥10 % H_2_O (*v*/*v*), which indicated that the minimum *a*_w_ required for HLADH catalytic activity in glyceline/water was 0.2 in this reaction system. This phenomenon is consistent with previous studies, which determined that the *a*_w_ at which oxidoreductases displayed 10 % of their maximal activity was 0.1–0.7.^27^ To the best of our knowledge, this is the first example reported in which the *a*_w_ concept is related to DES‐based biocatalytic systems. As non‐conventional media, DESs seem to follow the same premises of *a*_w_ as that of other previously reported solvents.

The high viscosity of DESs is considered to be a hurdle for their application as reaction media. Herein, water can be added to the reaction medium as a cosolvent to significantly lower the viscosity of mixtures of DES/water.6b, 28 Remarkably, a certain amount of water (up to 20 % *v*/*v*) can reduce the viscosity of DESs to the range of water, thus maintaining the nature of DESs as non‐conventional media (i.e., high solubility for hydrophobic substrates).6b, 29 This enables the setup of continuous processes with mixtures of DES/water with low viscosity,^30^ leading to highly attractive synergies for sustainable chemistry.^31^ The addition of water (up to 20 % *v*/*v*) led to an almost linear decrease in the viscosity of a mixture of glyceline/water (Figure S4). In any case, the viscosity (53 mPa s) of the mixture with 20 % (*v*/*v*) water was still much higher than that of pure buffer system (2 mPa s). The mass transfer limitation caused by the relatively high viscosity of the mixture may also account for the lower conversion than that of the pure buffer system.^32^ This is also supported by the observation that the reaction performed in a mixture of glyceline/water with 20 % (*v*/*v*) water at a shaking speed of 1200 rpm gave 10 % higher yield than that obtained at 900 rpm (data not shown).

For an in‐depth characterization of the protein–solvent interactions, MD simulations of HLADH in mixtures of glyceline/water (0–20 and 100 %, *v*/*v*) corresponding to those used in experiments were performed. After equilibrating the systems, 100 ns simulations in the NPT ensemble were performed for systems consisting of one HLADH protein solvated with the experimentally used glyceline/water concentrations (Figure S10). The last 40 ns of all simulations showed a diminishing energy drift; hence, this part of the trajectory was used for analysis.

**Figure 3:**
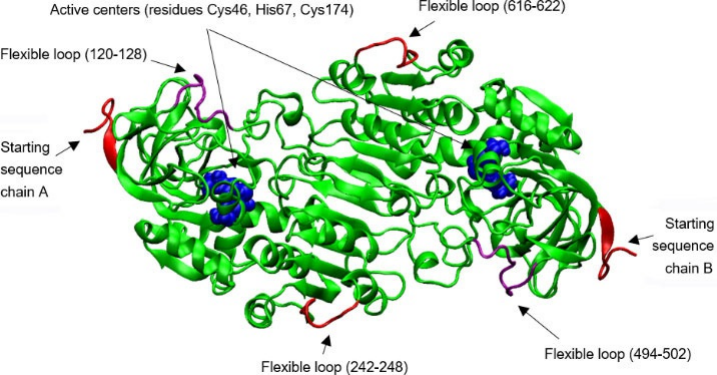
Illustration of the dimeric HLADH structure (PDB ID: 1HEU^33^). The amino acids of both active centers are illustrated as van der Waals spheres in blue. The flexible loops omitted from the RMSD and RMSF (see below) calculations are displayed in red and the loops also omitted from calculations in pure water are highlighted in purple.

To study the influence of different water contents on the structure of the enzyme, root‐mean‐square deviations (RMSDs) of the C_α_ atoms of HLADH were calculated with respect to the crystal structure coordinates (PDB ID: 1HEU).^33^ The residues at the start of the amino acid sequences (1–9 and 375–383), as well as loops from residues 242–248 and 616–622, were omitted in the RMSD calculations due to their high flexibility (Figure 3). The decision to omit these loops was based on an analysis of the residue‐wise root‐mean‐square fluctuations (RMSFs).18a For simulations in pure water, loops consisting of residues 120–128 and 494–502 were also very flexible, and therefore, excluded from the calculations. The RMSD values for different water contents show that the HLADH structure in glyceline/water is much closer to the crystalline structure than that of the purely aqueous case (Figure S12). The minimal RMSD occurs at 5 % (*v*/*v*) water, followed by a sharp increase between 10 and 12.5 % (*v*/*v*) water. The RMSD in the purely aqueous environment is up to three times larger than that investigated in mixtures of glyceline/water. The large error bars of more than 10 % in the purely aqueous system indicate that HLADH changes its structure more rapidly than that in lower water volume fractions. This is additionally supported by the results given in Figure S13, in which the RMSD was monitored over the simulation time and rapid fluctuations of the RMSD indicated structural transformations of the enzyme.

**Figure 4:**
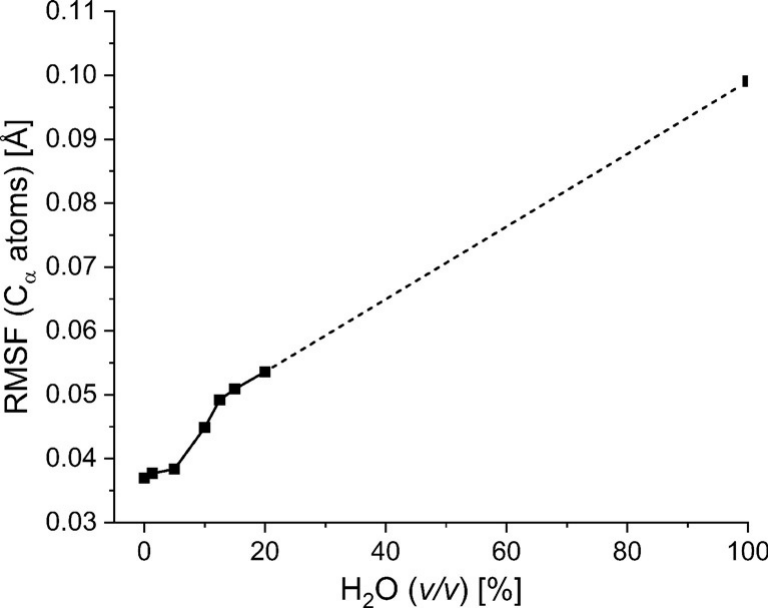
Average RMSFs of the C_α_ atoms of HLADH obtained in the simulations. The enzyme is solvated with mixtures of glyceline and water at 25 °C and 1 bar, which corresponds to the experimental setup. The data points are connected by solid and dashed lines to guide the eye.

Furthermore, the conformational flexibility of an enzyme is another important factor because it is essential for the catalytic activity. In an organic solvent, enzymes usually show a higher rigidity compared with that in an aqueous environment.18a, 18d The flexibility of HLADH in the respective mixtures of glyceline/water could be quantified by calculating the RMSF of the $C_{\alpha }$ atoms. Averages for the RMSF over all residues of HLADH are illustrated in Figure 4. The RMSF of HLADH at water contents below 10 % (*v*/*v*) indicate a rigid enzymatic structure relative to that in the purely aqueous system. This behavior is followed by an increase in enzymatic flexibility at 10 % (*v*/*v*) water; however, the RMSFs of mixtures of glyceline/water (12.5 to 20 % *v*/*v*) are still much lower than that of the average RMSF of HLADH in water.

The increase in the RMSD at 12.5 % (*v*/*v*) water indicates a modification of the enzyme structure, which correlates with an increase in the experimentally observed product yield. Hence, the onset of the enzymatic activity at 10 % (*v*/*v*) water (Figures 2 and S7) may be explained by an increase in the molecular flexibility of the enzyme.

Following the flexibility analysis of CALB performed by Trodler and Pleiss,18a the DES system should represent a favorable environment, in terms of conformational flexibility, because both DES components have negative octanol/water partition coefficients (log *P*(ChCl)=−3.77^34^ and log *P*(Gly)=−1.76^35^). Because DESs are highly hygroscopic, a low apparent log *P* of the DES mixture should also be expected. However, the results of this work are not coherent with the findings of Trodler and Pleiss, thus indicating that log *P* cannot be used as the only criterion to explain the flexibility of the enzyme. Both structural properties indicate that the solvent‐induced conformational changes of the enzyme, as well as its increased flexibility, may initiate the activity of the enzyme. These structural changes are supported by the results shown in Figure S17, which reveals the intraprotein hydrogen bonds. At water contents of <10 % (*v*/*v*), a decrease in the number of intraprotein hydrogen bonds was found, followed by a plateau between 10 and 20 % (*v*/*v*) water. Compared with the simulation in pure water, the number of hydrogen bonds of HLADH in the mixture of glyceline/water was up to 16 % higher. However, the role of the solvent molecules in this transformation, in particular, of water, still needs to be explored further.

**Figure 5:**
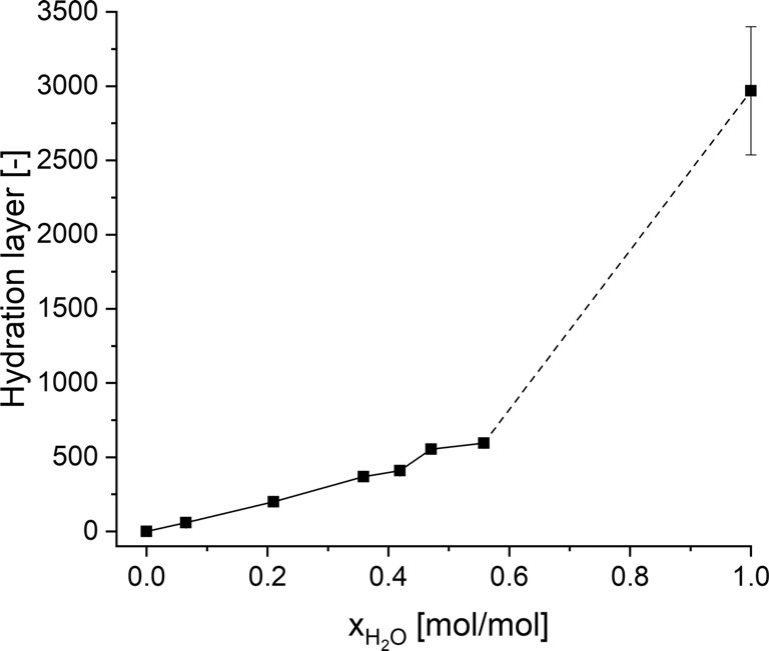
Hydration layer of HLADH in simulations of mixtures of glyceline and water versus the water mole fraction. Water, the oxygen atom of which is within 3.5 Å of any non‐hydrogen atom of HLADH, is considered to be in the hydration layer. Simulations were performed for one HLADH molecule in the respective mixture of glyceline/water at 25 °C and 1 bar. The data points are connected by solid and dashed lines to guide the eye.

Next to the structural properties of the protein, the hydration layer of the enzyme (i.e., the number of water molecules bound to the surface of the enzyme) is another important parameter that influences the activity and stability of the enzyme in nonaqueous media. A water molecule is considered to be in the first hydration layer, if its oxygen atom is within 3.5 Å of any non‐hydrogen atom of HLADH.18d, 36 The time averages for the first hydration layer of HLADH versus the water mole fraction are illustrated in Figure 5. A similar trend to that of water activity of the bulk phase could be found (Figure S3) because the hydration layer did not increase linearly with the water content. The correlation of molar versus volume water fractions is given in Figure S11 and Table S3. This indicates that the attractive interactions between water and glyceline compete with the interactions with the protein surface, which results in a preferential solvation of water in the DES bulk phase, rather than in hydration of the protein surface. This is in agreement with a study by Micaêlo and Soraes,18b who showed that the hydration level increased more for nonpolar solvents, such as hexane, than that for polar solvents. On the other hand, ionic and highly polar components of glyceline are able to mimic the hydrogen‐bond interactions of water, and therefore, compete with water for the polar and charged regions of HLADH. This resulted in a stripping of water from the surface of the enzyme, which was also observed in the case of different enzymes for other polar solvents.18a, 18b In addition, no evidence for a complete water layer around the protein in the DES mixtures could be found; hence, the enzyme was in direct contact with the glyceline molecules, even at 20 % (*v*/*v*) water. This means that the structural and catalytic behavior of HLADH is essentially influenced by the interactions with the DES. Combined with knowledge about the enzymatic activity (Figures 2 and S7), a hydration layer of 370±10 water molecules can be estimated as necessary to ensure the conformational flexibility, and hence, activity of the enzyme. This could be reached at 10 % (*v*/*v*) water (corresponding to 0.36 mol/mol water). However, it is yet to be shown if this minimal hydration layer, which is needed for enzymatic activity of HLADH, is similar for different solvents.

**Figure 6:**
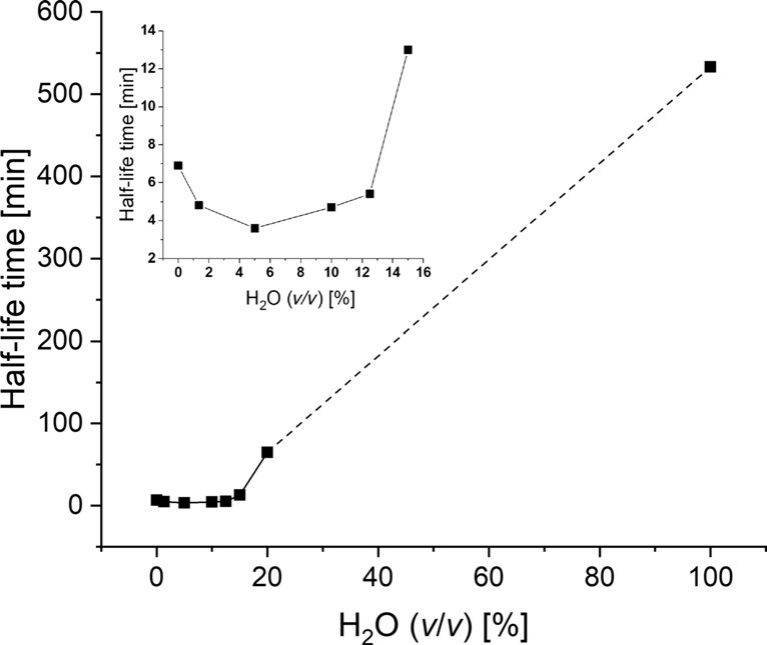
Half‐life of HLADH (1 mg mL^−1^) in a mixture of glyceline/water with water contents of 0–20 and 100 % (*v*/*v*) at 60 °C. The data points are connected by solid and dashed lines to guide the eye.

Subsequently, we evaluated the stability of HLADH in different mixtures of glyceline/water. As shown in Figure 6, the overall trend is that the half‐life of HLADH at 60 °C increased with increasing water content in the mixtures of glyceline/water. The temperature used in the activity assays was 25 °C, whereas the stability measurements were conducted at 60 °C (otherwise, the time range needed for an overview would have been days). The shortest half‐life of HLADH occurred at 5 % (*v*/*v*) H_2_O, followed by a sharp increase from 12.5 % (*v*/*v*) H_2_O. This observation coincides with the inflection temperature (*T*_i_; which represents the unfolding transition(s) or discrete changes in the structural integrity of a protein and can be used for comparing thermal stabilities of a protein) of HLADH in glyceline/water systems (Figures S5 and S6). The half‐life of HLADH in a purely aqueous system (533 min, ≈9 h) is approximately three orders of magnitude longer than those in glyceline with 0 to 12.5 % (*v*/*v*) H_2_O (between 4 and 7 min). In general, glyceline had a detrimental effect on the thermal stability of HLADH, which was consistent with the observation reported in the case of a purified KRED in mixtures of glyceline/water.^10^ However, many choline‐based DESs were found to stabilize heme‐dependent enzymes, such as horseradish peroxidase and cytochrome c peroxidase.7b, 7d These benefits could be attributed to perturbations in the heme microenvironment, which were demonstrated by UV/Vis and circular dichroism spectroscopic studies.

## Conclusion

This study has evaluated the activity (through initial rate measurements and product formation analyses) and stability (through half‐life measurements and *T*_i_ value determinations) of HLADH in mixtures of glyceline/water with water contents ranging from 0 to 20 % (*v*/*v*). A pure water system was also analyzed as a control. Our experimental evaluations also considered *a*_W_ values and the viscosity of mixtures of glyceline/water to obtain a better overview of these parameters. In parallel to our experimental studies, we have performed MD simulations to quantify the molecular flexibility of HLADH, the hydration layer on the surface of the enzyme, and intraprotein hydrogen bonds in different mixtures of glyceline/water. Overall, our empirical data and computational analyses could give a general overview, as summarized below.

The *a*_W_ value may give a good estimate of the protein activity because it determines the water hydration layer, and therefore, the flexibility of the enzyme. Although the exact influence of *a*_W_ on the HLADH activity and stability in different solvents is yet to be shown, it has potential to guide further investigations. Based on estimates, our results showed that a minimum *a*_W_ of 0.2 was needed for catalytic activity. Solutions of ChCl‐based DES with a low water content may not represent a favorable environment for HLADH.

Due to strong attractive interactions of water with glyceline, our results indicated that water could not sufficiently hydrate the enzyme at low water contents (<10 % *v*/*v*, in this system), which remained solvated in the mixture of glyceline/water. To increase the flexibility of HLADH, higher water contents, namely, ≥10 % (*v*/*v*), were necessary in the case of glyceline. However, the characteristic hydrogen‐bond structure of glyceline did not remain intact at high water contents, which resulted in the individual hydration of every DES component.^37^

Overall, this study represents the first detailed evaluation of experimental data on the catalytic performance of an oxidoreductase, together with MD simulations, in a holistic manner. We will explore other DESs to understand the effect of mixtures of DES/water in EC1‐catalyzed reactions in our future studies.

## Experimental Section

Details of the experimental data are provided in the Supporting Information. All MD simulations within this study have been performed by using the software package GROMACS version 2018.6.^38^ The OPLS‐DES force field^39^ and the TIP3P force field^40^ have been used for the DES glyceline and water, respectively. The combination of these force field models has been tested to reproduce the density of glyceline/water mixtures appropriately (Figure S9). The interactions of the protein molecule have been modeled with the OPLS‐AA/M force field.^41^ Details of the simulation procedure can be found in the Supporting Information.

## Conflict of interest

*The authors declare no conflict of interest*.

## Supporting information

As a service to our authors and readers, this journal provides supporting information supplied by the authors. Such materials are peer reviewed and may be re‐organized for online delivery, but are not copy‐edited or typeset. Technical support issues arising from supporting information (other than missing files) should be addressed to the authors.

Supplementary
