# Supplementary material for: Modeling Alcohol Dehydrogenase Catalysis in Deep Eutectic Solvent/Water Mixtures
Source: Chembiochem. 2019 Dec 13;21(6):811–7. doi: 10.1002/cbic.201900624 (PMC7154551; doi:10.1002/cbic.201900624)
Supplement: Supplementary file 1 — Supplementary [file CBIC-21-811-s001.pdf]

CHEMBIOCHEM

Supporting Information

Modeling Alcohol Dehydrogenase Catalysis in Deep Eutectic Solvent/Water Mixtures

Lei Huang+,[a] Jan Philipp Bittner+,[b] Pablo Domínguez de María,[c] Sven Jakobtorweihen,*[b] and Selin Kara*[a]

cbic_201900624_sm_miscellaneous_information.pdf

1. Materials

Chemicals, cultivation media components, and reagents were purchased from Sigma–Aldrich (St. Louis, USA), Carl Roth (Karlsruhe, Germany), VWR (Radnor, US) and used as received. Ni-NTA affinity resin was ordered from Expedeon (Cambridgeshire, UK) and BCA protein quantification kit (PierceTM) was obtained from Thermo Scientific (Rockford, USA). The recombinant pET-28b plasmids containing HLADH gene was from Dr. Diederik Johannes Opperman (University of Free State, South Africa).

2. Preparation of lyophilized purified HLADH

HLADH was heterologous expressed with pET28b(+) in *E. coli* BL21 (DE3). The preculture was grown on 20 mL LB medium containing 100 μg/mL kanamycin in 100 mL baffled flask at 37°C and 120 rpm overnight (~ 16 h). After which, 10 mL (2.5% v/v) of the preculture was used to inoculate 400 mL LB medium containing 100 μg/mL kanamycin in 2 L baffled flask, which was then incubated at 37°C and 120 rpm. After OD_{600} reached 0.6–0.8, IPTG was added to a final concentration of 0.5 mM and the incubation was continued at 24°C for 24 h. The cells were harvested by centrifugation at 8,000 rpm (5238 × g) at 4°C for 10 min. The obtained cell pellets were re-suspended in lysis buffer (50 mM NaH_2PO_4, 300 mM NaCl, 10 mM imidazole, pH 8.0) in a ratio of 40 mL lysis buffer to 20 g wet cells. The re-suspended cells were disrupted by ultrasonication (Sartorius Labsonic M with MS 73 probe) on ice at 60% amplitude and 0.4 cycle for 4 times of 4 min. The soluble protein was separated from the cell debris by centrifugation at 15,000 rpm (32735 × g) for 30 min at 4°C.

The obtained clear cell free extract (CFE) was applied twice to the Ni-NTA column, which was previously equilibrated with 5 column volumes (CV) of wash buffer (50 mM NaH_2PO_4, 300 mM NaCl, 20 mM imidazole, pH 8.0). The column was then washed with 10 CV of wash buffer. Next, 3 CV of elution buffer (50 mM NaH_2PO_4, 300 mM NaCl, 250 mM imidazole, pH 8.0) was added to the column to elute the bound target enzyme. 5 μL of samples were collected from each wash- and elution fraction for subsequent SDS-PAGE analysis. For the removal of imidazole, the column was washed with the following buffers: 1) 5 CV of wash buffer, 2) 3 CV of MES buffer, 3) 5 CV of deionized water, 4) 3 CV of 30% ethanol.

All the fractions containing the purified HLADH based on the SDS-PAGE analysis (**Figure S1**) were collected for the further ultrafiltration using centrifugal unit (Millipore, 10 kDa, 1389 × g) at 4°C. The concentrated enzyme solutions were diluted with desalting buffer (10 mM Tris-HCl, pH 7.5) twice to approximate 100 times dilution and

finally concentrated to 5.0 mL. The obtained purified HLADH was firstly frozen at -80°C for overnight and subsequently freeze-dried at 0.03 mbar and -60°C for 40 h.

5.0 mL of purified HLADH with the protein concentration of 23 mg/mL was obtained from 21.4 g wet cells. After the lyophilization, 112 g lyophilized HLADH was obtained, thus the productivity is 5.23 mg protein/g cells (**Figure S1**).

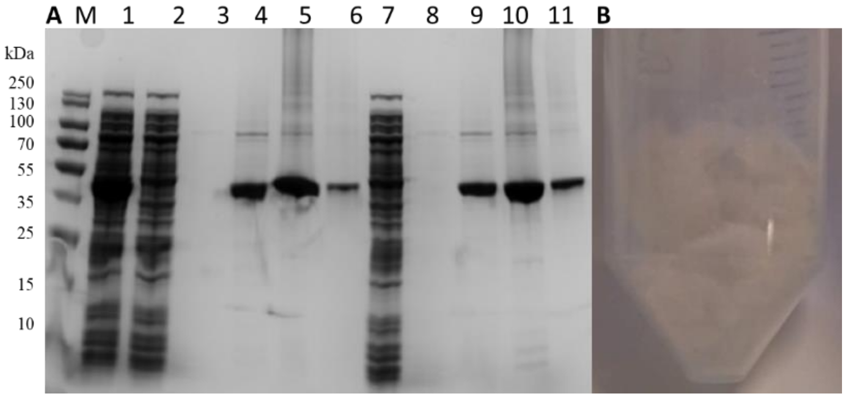

Figure S1. (**A**) SDS-PAGE analysis of HLADH purification samples. M: Maker, lane 1: CFE, lanes 2 and 7: flow through, lanes 3 and 8: washing fractions, lanes 4–6 and 9–11: elution fractions. (**B**) Lyophilized purified HLADH.

3. Synthesis of glyceline

Choline chloride (ChCl) and glycerol (Gly) were directly weighed in a flask in a molar ratio of 1:2. Then, the mixture was heated and stirred (IKA Magnetic stirrer, 250 rpm) at 80°C until a colorless liquid was formed (normally less than one hour).

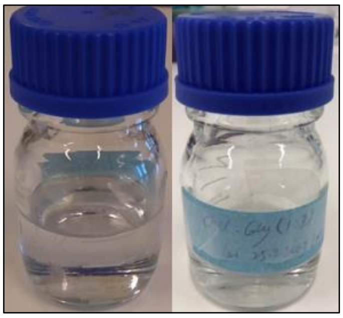

Figure S2. The appearance of synthesized glyceline.

4. Determining the water activity (*a*$_w$) of glyceline-water binary mixtures

Binary mixtures (5 mL) of glyceline with various water contents of 0–20% (v/v) and 100% (v/v) were freshly prepared and incubated at 60°C for 1 hour in 25 mL sealed glass bottles. The thermodynamic water activity (a_w) of these mixtures were then determined at room temperature (24–25°C) using HMT337 Humidity and Temperature Transmitter (vaisala, Vantaa, Finland). Here, the a_W was measured based on the ratio of the water vapor pressure in the glyceline-water mixtures (p) to the vapor pressure of pure water (p_0) ($a_w = p/p_0$).

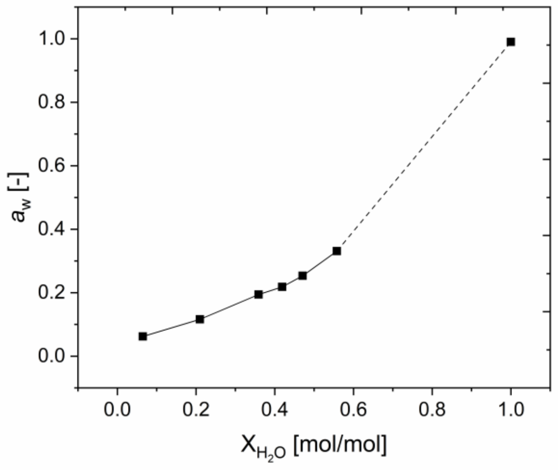

Figure S3. Thermodynamic water activity (a_w) of glyceline-water mixtures as a function of water content in glyceline based on mole fraction. The data points are connected by a solid line and a dash line to guide the eye.

5. Determining the viscosity of glyceline-water binary mixtures

The dynamic viscosity (η) of glyceline-water mixtures was measured on 2 mL sample with a Brookfield Digital Rheometer (Model DV-III Ultra, Brookfield Engineering Laboratories Inc., MA, USA) equipped with a spindle CPE41, at different shear rates between 0.2 s^{-1} and 500 s^{-1} at room temperature (24–25°C).

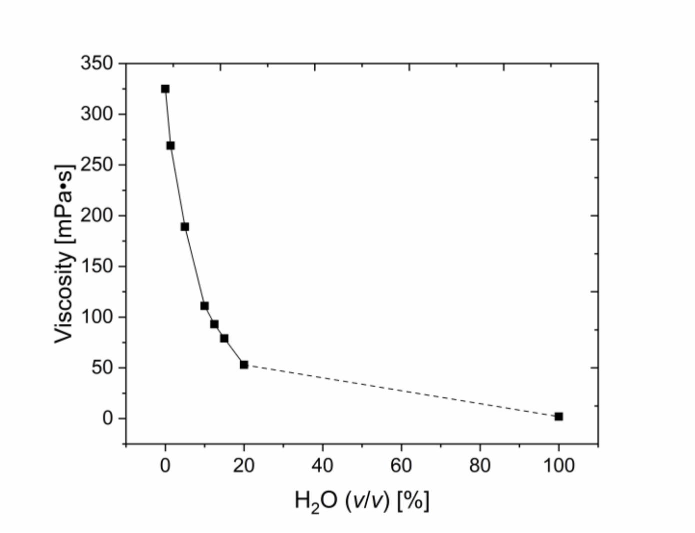

Figure S4. The viscosity (η) of glyceline-water mixtures as a function of water content. The data points are connected by a solid line and a dash line to guide the eye.

6. Determining the T_i of HLADH in glyceline-water binary mixtures

Tycho NT.6 instrument (NanoTemper Technologies) was used to determine the inflection temperature (T_i) of HLADH with the concentration of 1.0 mg/mL in glyceline with various water contents. The Tycho NT.6 capillaries (high precision glass capillaries specifically developed for use with Tycho NT.6) containing 10 μL sample were placed on the sample holder. A temperature gradient of 30°C/min from 35°C to 95°C was applied and the fluorescence of intrinsic tryptophan and tyrosine of HLADH was recorded at 330 nm and 350 nm. Tycho utilizes a fast and defined thermal ramp to unfold HLADH and identifies the inflection temperature (T_i) that represent unfolding transition(s) or discrete changes in the structural integrity of HLADH and can be used for comparing thermal stabilities of HLADH in glyceline with various water content.

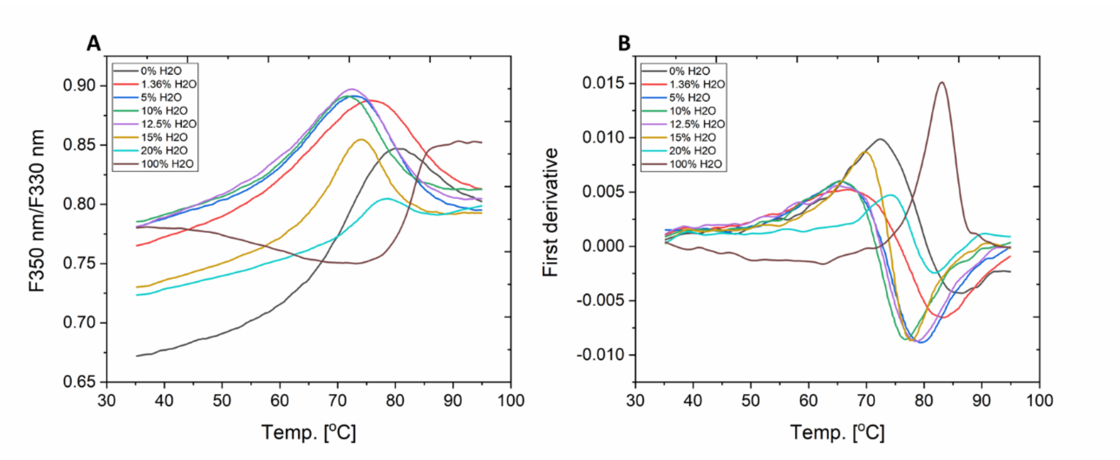

Figure S5. Analysis of HLADH inflection temperature (T_i) in the glyceline with various water content. (**A**) Thermal unfolding curves (**B**) Corresponding first derivative indicating inflection temperature (T_i) values.

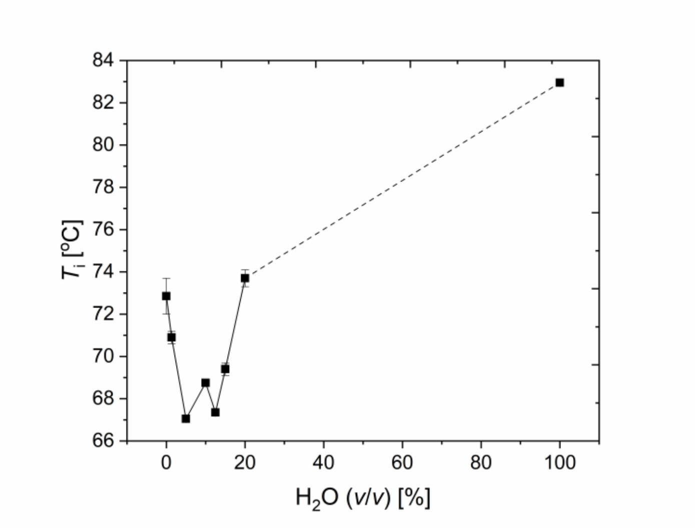

Figure S6. The inflection temperatures (T_i) of HLADH (1.0 mg/mL) in glyceline with various water contents, 0–20% (*v*/*v*) and 100% (*v*/*v*). The data points are connected by a solid line and a dash line to guide the eye.

7. Determining the $t_{1/2}$ of HLADH in glyceline-water binary mixtures

The half-life times of HLADH in various glyceline-water mixtures were determined by incubating 500 μL of 1.0 mg/mL purified HLADH at 60°C. The samples were taken at aliquot time points ranging from 0 to 20 h and the residual activities were detected at 25°C. The half-life times ($t_{1/2}$) were calculated based on **Equation 1**.

$$t_{1/2} = \frac{\text{Ln } 2}{k_{des}} \quad \text{(Eqn. 1)}$$

$t_{1/2}$: Half-life time [h]

k_{des}: Deactivation constant [h^{-1}]

8. General conditions for the HLADH-catalyzed reductions

The stock of NAD^+ (20 mM) and lyophilized purified HLADH (20 mg/mL) were prepared in Tris-HCl (50 mM, pH 7.5) and incubated at 25°C for 30 minutes. The stock of substrate cyclohexane (200 mM) and co-substrate 1,4-butanediol (100 mM) were prepared in glyceline and Tris-HCl (50 mM, pH 7.5), respectively. All the reaction components (cyclohexane, 1,4-butanediol, glyceline, water (in the form of Tris-HCl buffer), NAD^+ and HLADH) of each reaction system were applied in 1.5 mL GC vials based on the corresponding water contents (0%, 1.36%, 5%, 10%, 12.5%, 15%, 20%, and 100% v/v) and incubated at 25°C and 1200 rpm. The final each system had a total volume of 1.0 mL and contained 100 mM cyclohexane, 50 mM 1,4-butanediol, 1 mg/mL HLADH, 1 mM NAD^+ and various water contents. Each reaction was performed in duplicate. Aliquot samples were taken at definite time and analyzed with GC.

Table S1. Components of HLADH-catalyzed reaction in glyceline with various water content.

Water content [v/v]	Substrate stock in glyceline [μL]	Substrate stock in Tris-HCl [μL]	HLADH and NAD^+ stock [μL]	Glyce line [μL]	Tris-HCl [μL]	HLADH (lyophilized) [mg]	NAD^+ (powder) [mg]
0%	500	0	0	500	0	1	0.77 (1 mM)
1.36%	500	0	0	486.4	13.6	1	0.77 (1 mM)
5%	500	0	50	450	0	0	0
10%	500	0	50	400	50	0	0
12.5%	500	0	50	375	75	0	0
15%	500	0	50	350	100	0	0
20%	500	0	50	300	150	0	0
100%	0	500	50	0	450	0	0

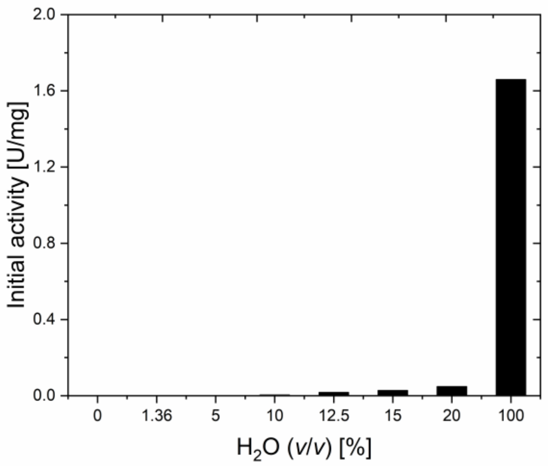

Figure S7. Initial activity of HLADH-catalyzed reduction in glyceline with various water contents (based on the data at 0.1 h while all product formation less than 10%). Reaction conditions: 100 mM CHO, 50 mM 1,4-BD, 1 mM NAD^+, 1 mg/mL purified HLADH in glyceline-H_2O media at 25°C and 1200 rpm. Buffer (50 mM Tris-HCl, pH 7.5) was added to incubate the enzyme with the NAD^+.

9. Gas chromatography analysis

Aliquots samples (50 µL) from each reaction system were taken at definite time intervals and mixed with 250 µL of ethyl acetate (2 mM methyl benzoate as the internal standard). After centrifuging (13,000 rpm; 1 min) and separating the two phases, the EtOAc layer was dried with anhydrous $MgSO_4$. All reaction components were then analyzed by gas chromatography (GC) and the methods were developed with ß-DEX 120 column (30 m x 0.25 mm x 0.25 µm, Supelco® Analytical, USA; catalogue reference: 24304). Peaks were identified by standards. The details could be seen below in **Table S2**. The concentrations of target components were calculated based on the corresponding GC calibration in each reaction system.

Table S2. Details of GC method used in this study.

Heating program			Column	Components	t_R [min]
Rate [°C/min]	T [°C]	Hold [min]	ß-DEX 120 30 m x 0.25 mm x 0.25 µm	CHO	13.440
-	70	5		CHL	14.397
20	140	7		GBL	15.074
20	160	4		MB	17.463
20	220	0		1,4-BD	18.604

T(Injector): 250°C; Detector: FID; *T*(Detector): 250°C; Carrier gas: He; Pressure: 0.366 bar
Column flow: 0.4 mL/min; Total flow: 11.4 mL/min; Split ratio: 30

CHO: cyclohexanone, CHL: cyclohexanol, GBL: γ-butyrolactone, MB: methyl benzoate, 1,4-BD: 1,4-butanediol.

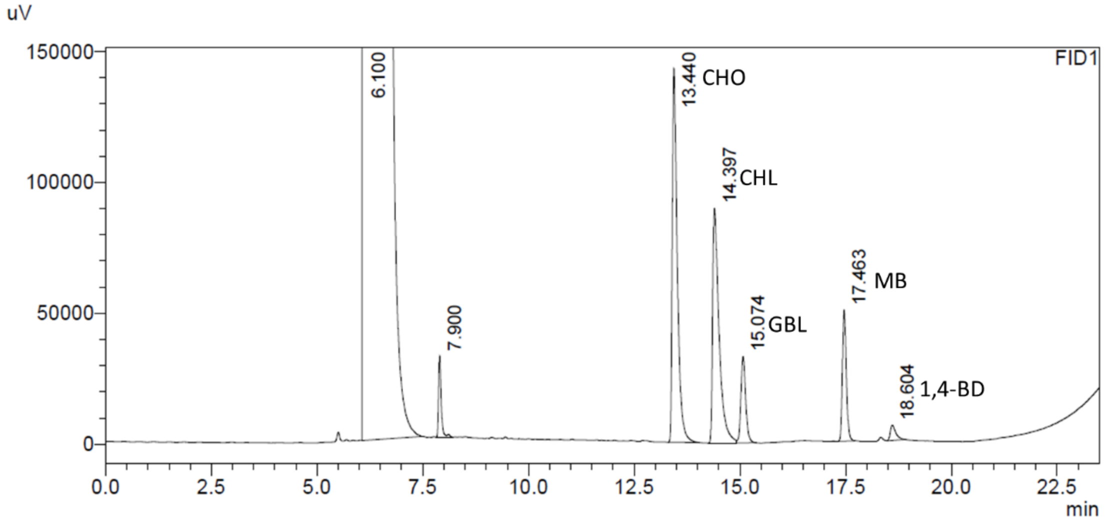

Figure S8. Example of a GC chromatography spectrum. CHO: cyclohexanone, CHL: cyclohexanol, GBL: γ-butyrolactone, MB: methyl benzoate (internal standard), 1,4-BD: 1,4-butanediol.

10. Force field for MD simulations of biomolecules

For MD simulations a force field model is needed, which describes the interatomic and intra-atomic interactions between the simulated molecules. Prior to the application of MD simulations to large protein systems the validation of the used force field is of great importance. In particular, the application of the novel solvent group of DESs requires a careful validation of the force field parameters.

The recently published OPLS-DES force field by Doherty and Acevedo[1] was tested to reproduce the density of mixtures of choline chloride with varying water content. Therefore, MD simulations of pure glyceline as well as in mixtures with a water content

up to 47% (mol/mol) using the software package GROMACS version 2018[2] were compared to an experimental density correlation.[3] Compared to the publication of Doherty and Acevedo,[1] the simulation parameters have been tuned to be better suitable for simulations containing a protein considering the parameters usually used for protein simulations and considering that the system is much larger. As all bonds between hydrogen and other atoms are fixed using the LINCS algorithm, a time step of 1 fs is not necessary. Instead a larger time step of 2 fs would be more efficient in particular for large system sizes. In addition to the time step the cut-off radius for the van-der-Waals and electrostatic interactions has been changed compared to Doherty and Acevedo.[1] The force field parameters of OPLS-DES have been fitted using a cut-off radius of 1.6 nm, whereas in the original OPLS-AA-force field, intended for protein simulations, it was set to 1.1 nm. Accordingly, the original cut-off of 1.1 nm was used in this work by smoothly switching the force between 0.9 and 1.1 nm. The adjustments mentioned above lead to an increase in the performance of the OPLS-DES force field by ca. 70%, importantly the quality of the results is only marginally changed (see below). The resulting densities of the choline chloride (ChCl)–glycerol (Gly) mixtures (**Figure S9**) are in quantitative agreement with the experimental correlation, as the deviations are within 1% of the experimental values. In addition, radial distribution functions (RDF) of pure choline chloride glycerol are in agreement with the results of Doherty and Acevedo (data not shown).[1]

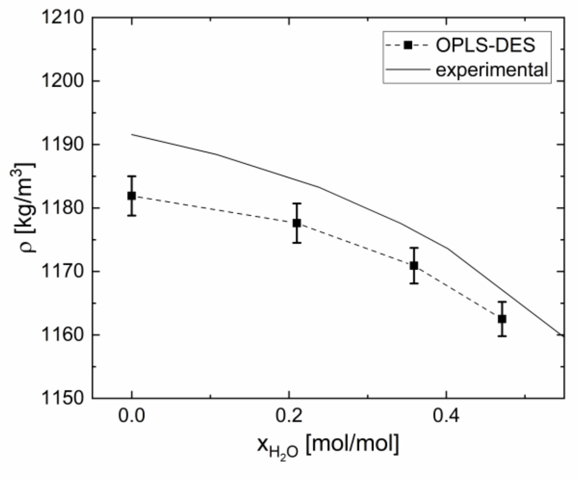

Figure S9. Liquid densities of glyceline-water mixtures at 1 bar and 298.15 K (25°C): simulated with the OPLS-DES force field[1] (black squares and dash line) and experimental correlation[3] (black solid line).

11. MD simulations procedure for HLADH in glyceline-water mixtures

Owing to large viscosities, MD simulations of DESs have proven to be challenging due to their slow dynamic behavior.[4] The challenges begin with the equilibration of the systems, which is largely influenced by the slow dynamics of most DES. In order to overcome this issue, Perkins et al.[4a, 4b] suggested to use compression and decompression scheme to ensure an efficient equilibration of highly viscous systems, which was priory applied for polymeric systems.[5] This scheme also later adopted by Mainberger et al.,[4c] was tested in this work for the MD simulations of HLADH in glyceline-water mixtures. However, the resulting simulations show large energy drifts, which occur even at long simulations times larger than 80 ns. Therefore, a different equilibration scheme based on temperature annealing is proposed to equilibrate the simulation boxes. A similar scheme has been applied by Liu et al. for ionic liquids.[6]

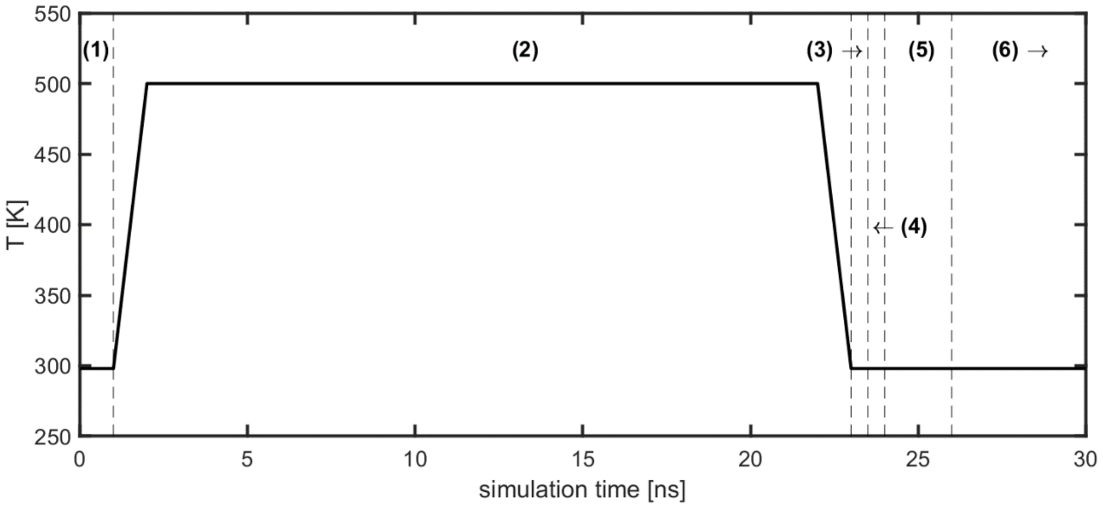

Figure S10. Equilibration procedure of the MD simulations of HLADH in glyceline mixtures following the energy minimization. (1) NVT constraining the positions of all enzyme atoms, $\mathrm{d}t = 1\mathrm{fs}$, (2) NVT with temperature annealing to 500 K, $\mathrm{d}t = 2\mathrm{fs}$ (3) NVT constraining only the protein backbone, $\mathrm{d}t = 1\mathrm{fs}$, (4) NVT constraining only the C_α-atoms of HLADH, $\mathrm{d}t = 1\mathrm{fs}$, (5) NPT equilibration without any position constrains and using the Berendsen barostat, $\mathrm{d}t = 2\mathrm{fs}$, and (6) simulations in the NPT ensemble using the Parrinello-Rahman barostat (continues for 100 ns), where the last 40 ns were used for analysis, $\mathrm{d}t = 2\mathrm{fs}$.

All MD simulations of this work were performed with the software package GROMACS version 2018.6.[2] The OPLS-DES force field[1] and the TIP3P force field[7] have been used for the DES glyceline and water, respectively. The protein interactions have been modeled with the OPLS-AA/M force field.[8] The temperature curve applied for the equilibrations is displayed in **Figure S10**. Whereby the used equilibration scheme for the protein systems consists of five consecutive steps. Starting with the

crystallographic structure of HLADH (PDB entry 1HEU) embedded in a solution using packmol[9], an energy minimization with the steepest decent algorithm was performed for 5000 steps. Afterwards, initial velocities for all molecules according to a Maxwell-distribution at 278 K are assigned and a 1 ns simulation at 298 K using a velocity rescale thermostat and by constraining the positions of all enzyme atoms has been performed. In order to ensure a proper mixing of the liquid around the constrained enzyme, the temperature is subsequently increased to 500 K during a period of 1 ns and then kept constant for 20 ns. A temperature of 500 K has been chosen as this is the maximal temperature the OPLS-DES force field was validated for. After cooling down the simulated system to 298 K during a period of 1 ns the enzyme structure is stepwise released from the crystal structure during two simulations of 0.5 ns in the NVT ensemble. Restraining the positions of the enzyme's atoms, backbone and C_α-atoms, corresponding to the three equilibration steps in the NVT ensemble, allows a smooth equilibration of the solvent molecules without disturbing the structure of HLADH. The release of the enzyme was followed by an NPT equilibration for 2 ns without any position restrains and using a Berendsen barostat for adjusting the system pressure to 1 bar.

The equilibration phase was followed by a simulation in the NPT ensemble for 100 ns, whereby the barostat was switched to Parrinello-Rahman, which reproduces the correct ensemble. The long simulation time was chosen in order to observe significant changes of the protein structure. All simulations show a diminishing energy drift for the last 40 ns of the simulations (data not shown), hence, this part of the trajectory was used for sampling the protein and solvent properties.

12. Conversion of volume fractions and mole fractions for glyceline water mixtures

In terms of molecular dynamics simulations, the experimentally used volume fractions need to be transferred to mole fractions in order to relate it to the MD simulations. A corresponding conversion plot can be found below (**Figure S11**), the concentrations investigated in this work are listed in Table S3. Whereby, the mole fractions x_i in the MD simulations have been calculated by the following equation:

$$x_i = \frac{N_i}{N_{\mathrm{water}} + N_{\mathrm{glycerol}} + N_{\mathrm{ChCl}}} \qquad \text{(Eqn. 2)}$$

In order to make sure that the same concentrations are used in the experiments and simulations the volume fractions ϕ_i have been converted to mole fractions via the density ρ_j and molar masses M_j.

$$x_i = \frac{\frac{\phi_i \cdot \rho_i}{M_i}}{\sum_j \frac{\phi_j \cdot \rho_j}{M_j}} \quad \text{(Eqn. 3)}$$

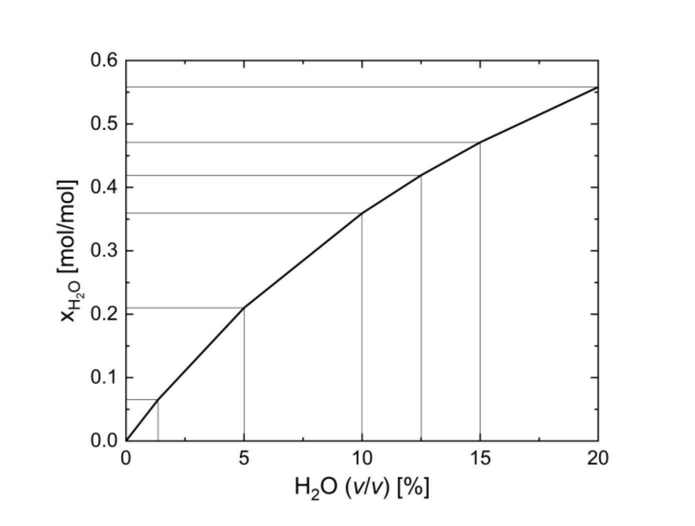

Figure S11. Dependency of the volume fractions of water on the water mole fractions for the investigated glyceline-water mixtures.

Table S3. Conversion between water volume fractions and mole fractions of water used in this work.

Volume fraction [%H_2O (v/v)]	Mole fraction [mol/mol]
0	0
1.36	0.065
5	0.210
10	0.359
12.5	0.419
15	0.471
20	0.558
100	1

13. Structural properties of horse liver alcohol dehydrogenase (HLADH)

13.1. Crystallographic structure of HLADH

As starting point for the MD simulation, the experimentally measured crystallographic structure of HLADH (PDB entry 1HEU) was used. A schematic illustration of the HLADH structure is displayed in **Figure 3**. In general, the enzyme can be divided into

two parts: the substrate binding domain (residues 1-175, 319-549, 693-748) and the coenzyme binding domain (residues 176-318, 550-692).[10] The coenzyme binding domain is located in the middle of the protein structure, whereas the substrate binding domain, which includes the active centers are located at the left and right site of the protein structure illustrated in **Figure 3**.

HLADH has a dimeric structure of two identical subunits connected via hydrogen bonds.[10-11] Hence, the enzyme owes two active centers in the middle of each monomer (**Figure 3**, highlighted in blue). Though, the three amino acids of each active center (Cys46, His67, Cys174) belong to one chain (for instance chain A), the substrate binding pockets also consists of amino acids of the other chain (in this example chain B). The amino acids along the substrate pocket of chain A, according to Eklund et al.[10-11] are summarized in **Table S4**. This means that HLADH is only functional in form of the dimeric structure, and not in terms of the single monomers. **Figure 3** also illustrates the flexible part at the start of both subunits as well as flexible loops omitted in the calculations of structural properties (242-248 and 616-622) in red. In case of pure water the loops consisting of the residues 120-128 and 616-622 (highlighted in purple) are also excluded from the calculations.

Table S4. Amino acids of the substrate binding pocket of chain A according to Eklund et al..[10-11]

Chain A		Chain B
Ser48	Phe140	Met306
Leu57	Leu141	Leu309
Val58	Thr178	Ser310
Phe93	Val294	
Phe110	Pro296	
Ser117	Ile318	

13.2. Root mean square deviations of the C_α-atoms of HLADH in different glyceline-water mixtures

The RMSD of the C_α-atoms of HLADH with respect to the crystallographic structure (PDB entry 1HEU) are monitored over the simulation time in order to check for structural changes of the enzyme in different environments. The development of the RMSD over the simulation time for the different water concentrations are illustrated in **Figure S12** as well as the time averages for the last 40 ns of the trajectory are shown in **Figure S13**.

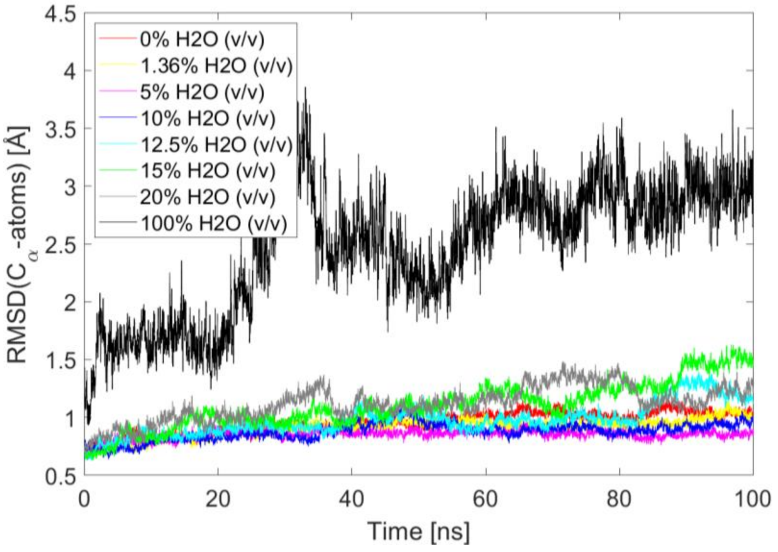

Figure S12. Root mean square deviations (RMSD) of the C_α-atoms of HLADH in the simulations compared to the crystal structure over the simulation time. The enzyme is solvated with mixtures of glyceline and water at 25 °C and 1 bar corresponding to the experimental set-up.

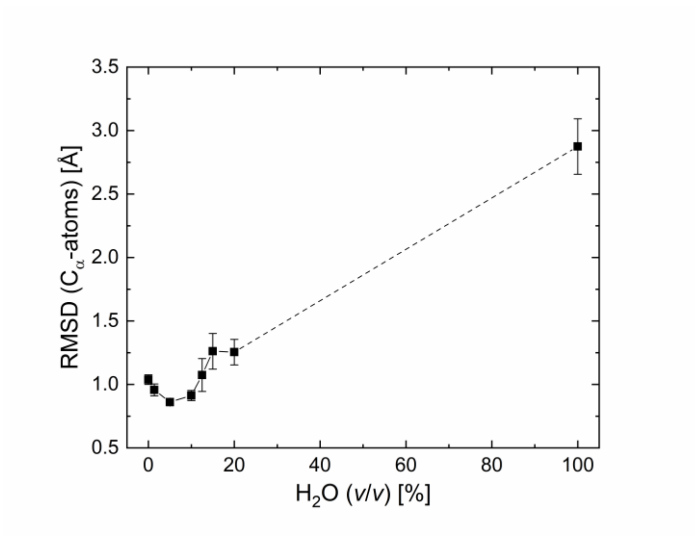

Figure S13. Root mean square deviations (RMSD) of the C_α-atoms of HLADH in the simulations compared to the crystal structure versus the water volume fraction. The enzyme is solvated with mixtures of glyceline and water at 25°C and 1 bar corresponding to the experimental set-up. The data points are connected by a solid line and a dash line to guide the eye.

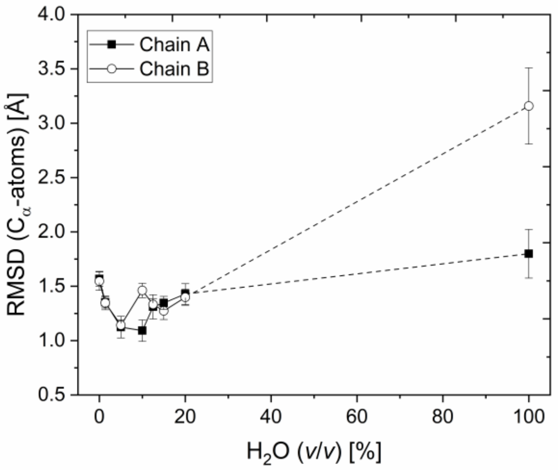

Figure S14. Root mean square deviations (RMSD) of the C_α-atoms of the binding site and pocket of HLADH in the simulations compared to the crystal structure. The enzyme is solvated with mixtures of glyceline and water at 25 °C and 1 bar corresponding to the experimental set-up. The data points are connected by solid lines and dash lines to guide the eye.

In addition, the structural changes for both active centers including the binding site pockets are illustrated in **Figure S14**. Besides the simulations in pure water and 10% (v/v) water the RMSD of the pocket and active center are similar for both chains. However, at 10% (v/v) the binding site of chain A is closer to the crystalline state compared to chain B. A similar trend could be found for the pure aqueous environment. This is counterintuitive, as the enzyme structure is consisting of two identical chains and is perfectly symmetric. An analysis of the minimal distance of the binding site and pocket residues to the solvent molecules revealed a closer contact to choline and chloride ions for chain A compared to chain B (**Figure S15**). The minimal distance to chloride was much lower than in all other cases, particularly compared to the pure glyceline case (data not shown). The strong interactions between chloride and the enzyme may lead to different structures, which could explain the differences observed in the simulations. This is particularly interesting as it coincides with the beginning of a catalytic activity. Nevertheless, this may not explain the huge difference for the pure water case, as both catalytic centers are equally hydrated in those simulations.

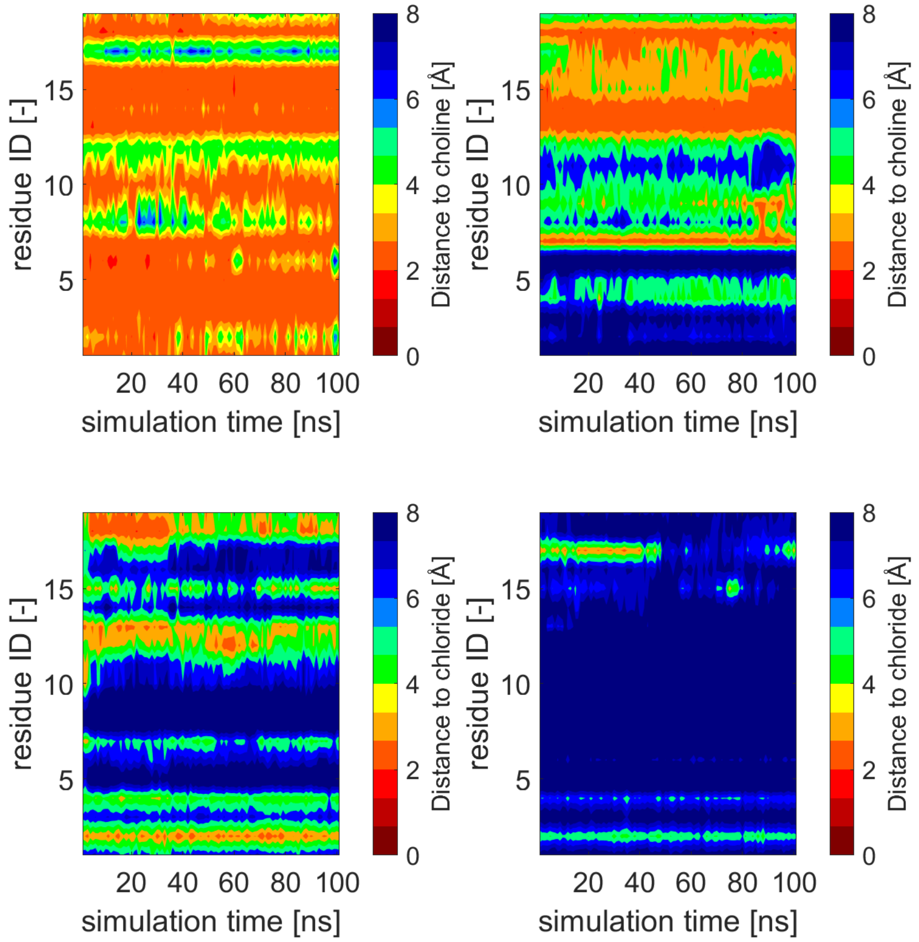

Figure S15. Minimal distance of choline (top) and chloride (bottom) to the binding site and pocket of chain A (left) and chain B (right) in the simulations monitored versus the simulation time. The residues along the binding site and pocket are sorted from the active site to the surface of the protein. The enzyme is solvated with mixtures of glyceline and 10% H_2O (*v*/*v*) at 25 °C and 1 bar.

13.3. Root mean square fluctuations (RMSF) per residue of the C_α-atoms of HLADH in different glyceline-water mixtures

An analysis of the RMSF per residue has been performed and the resulting values are illustrated in **Figure S16**. Based on this study the flexible loops from the residues 1-9, 375-38, 242-248 and 616-622 have been omitted from the average RMSF and RMSD calculations. For simulations in pure water, the loops of the residues 120-128 and 494-502 showed also high flexibilities and were therefore excluded from the average RMSD and RMSF calculations.

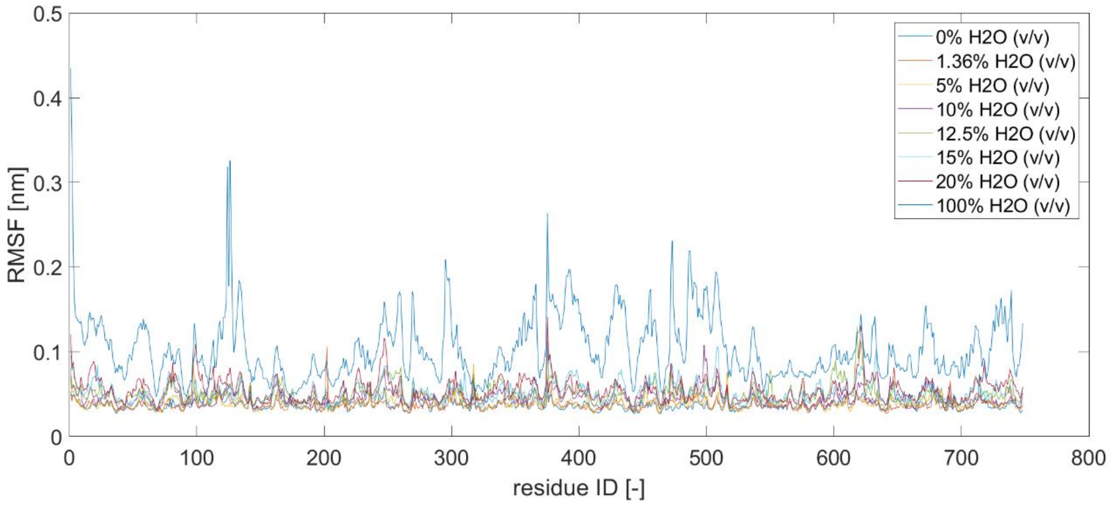

Figure S16. Root mean square fluctuations of the C_α-atoms of HLADH in gylceline water mixtures per residue. The enzyme is solvated with glyceline-water mixtures at 25°C and 1 bar corresponding to the experimental set-up.

14. Intra-protein hydrogen bonds

The intra-protein hydrogen bonds have been calculated with the gmx hbond tool implemented in the GROMACS package for HLADH in all investigated glyceline-water mixtures. Whereby, a cut-off distance of 0.25 nm and a cut-off angle of 30° have been used. The resulting values are shown in **Figure S17**.

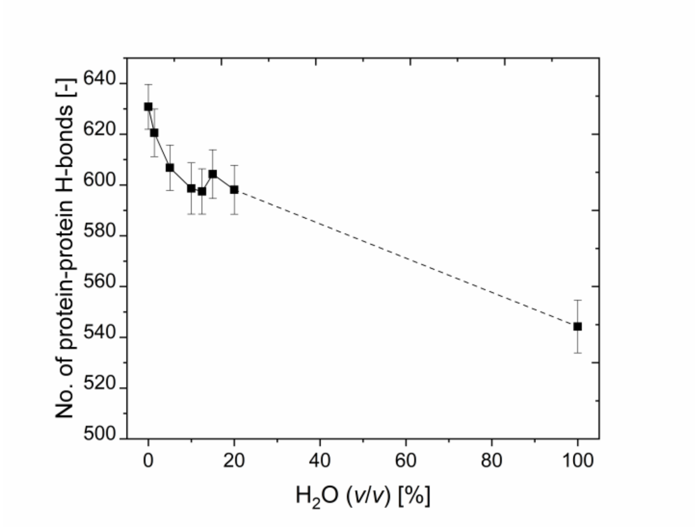

Figure S17. Intra-protein hydrogen bonds of HLADH in the simulations versus the water volume fraction. The enzyme is solvated with mixtures of glyceline and water at 25 °C and 1 bar corresponding to the experimental set-up. The data points are connected by a solid line and a dash line to guide the eye.

References

[1] B. Doherty, O. Acevedo, *J. Phys. Chem. B* **2018**, *122*, 9982-9993.

[2] M. J. Abraham, T. Murtola, R. Schulz, S. Páll, J. C. Smith, B. Hess, E. Lindahl, *SoftwareX* **2015**, *1*, 19-25.

[3] A. Yadav, S. Trivedi, R. Rai, S. Pandey, *Fluid Phase Equilib.* **2014**, *367*, 135-142.

[4] a) S. L. Perkins, P. Painter, C. M. Colina, *J. Phys. Chem. B* **2013**, *117*, 10250-10260; b) S. L. Perkins, P. Painter, C. M. Colina, *J. Chem. Eng. Data* **2014**, *59*, 3652-3662; c) S. Mainberger, M. Kindlein, F. Bezold, E. Elts, M. Minceva, H. Briesen, *Mol. Phys.* **2017**, *115*, 1309-1321.

[5] G. S. Larsen, P. Lin, K. E. Hart, C. M. Colina, *Macromolecules* **2011**, *44*, 6944-6951.

[6] H. Liu, E. Maginn, A. E. Visser, N. J. Bridges, E. B. Fox, *Ind. Eng. Chem. Res.* **2012**, *51*, 7242-7254.

[7] W. L. Jorgensen, J. Chandrasekhar, J. D. Madura, R. W. Impey, M. L. Klein, *J. Chem. Phys.* **1983**, *79*, 926-935.

[8] M. J. Robertson, J. Tirado-Rives, W. L. Jorgensen, *J. Chem. Theory Comput.* **2015**, *11*, 3499-3509.

[9] L. Martínez, R. Andrade, E. G. Birgin, J. M. Martínez, *J. Comput. Chem.* **2009**, *30*, 2157-2164.

[10] H. Eklund, B. Nordström, E. Zeppezauer, G. Söderlund, I. Ohlsson, T. Boiwe, B.-O. Söderberg, O. Tapia, C.-I. Brändén, Å. Åkeson, *J. Mol. Biol.* **1976**, *102*, 27-59.

[11] H. Eklund, B. Plapp, J. Samama, C. Brändén, *J. Biol. Chem.* **1982**, *257*, 14349-14358.
